# Bone flap resorption after autologous cranioplasty for traumatic brain injury: a case–control study in Vietnam

**DOI:** 10.1007/s00701-026-06993-2

**Published:** 2026-08-14

**Authors:** Huy Ngoc Pham, Nguyen Cong Tien Anh, Linh Khanh Nguyen, Son Hong Ha, Dinh Van Tran, Tuan Anh Le, Ha Dai Duong, He Van Dong

**Affiliations:** 1 Department of Neurosurgery 1, Viet Duc University Hospital, Hanoi, Vietnam; 2 Department of Surgery, Hanoi Medical University, Hanoi, Vietnam

**Keywords:** Cranioplasty, Bone resorption, Autologous bone, Traumatic brain injury, Risk factors

## Abstract

**Introduction:**

Autologous bone is a commonly used material for cranioplasty following decompressive craniectomy; however, it is accompanied by the inherent risk of resorption. This study aimed to identify factors associated with bone flap resorption (BFR) in patients undergoing autologous cranioplasty after decompressive craniectomy for traumatic brain injury.

**Methods:**

A matched case–control study was conducted at Viet Duc University Hospital between January 2022 and July 2025. Cases were defined as patients who developed severe BFR requiring revision surgery. Each case was matched with two controls without BFR based on cranioplasty date and follow-up duration. The association of demographic, general health, trauma history and peri-cranioplasty characteristics with BFR was assessed using conditional logistic regression.

**Results:**

A total of 71 cases and 142 matched controls were included in this study. BFR that required surgical revision occurred predominantly within two years after cranioplasty (90%). The mean time interval from cranioplasty to complication diagnosis was 444 ± 212 days. Large-sized bone flap (OR: 3.96, 95% CI: 1.86—8.43), fragmented bone flap (OR: 7.84, 95% CI: 1.38—44.53), and chronic viral hepatitis (either B or C) (OR: 4.32, 95% CI: 1.54—12.15) were independent associated factors of BFR. Young age (< 18), frontal sinus fracture, and ventriculoperitoneal shunt implantation showed a trend to increase risk of BFR but failed to reach statistical significance in multivariate analysis.

**Conclusions:**

In our study, bone fragmentation, large bone flap size, and chronic viral hepatitis were associated with increased risk of bone flap resorption after autologous cranioplasty in TBI patients. Further studies are necessary to confirm these findings, especially concerning the role of chronic viral hepatitis.

## Introduction

Bone flap resorption is a concerning complication in autologous cranioplasty (CP) following decompressive craniectomy (DC). It causes thinning, weakening or even complete lysis of the flap, thereby compromising skull integrity and possibly necessitating revision surgery. A study in Finland found that up to 90% of cases experienced BFR to some extent with a mean follow-up of 52 months [28] while other studies reported around 20% of patients developing severe BFR [5, 8–11, 14, 31, 36]. While decompressive craniectomies are sometimes performed in cases involving strokes and tumors, traumatic brain injuries (TBI) remain the most common cause. Additionally, higher rates of post-cranioplasty BFR are found in the TBI group compared to stroke or tumor cases [23, 41].

Several factors predisposing patients to bone necrosis have been suggested such as young age [5, 8, 10, 14, 28, 29], bone fragmentation [4, 5, 8–10], distance between the graft-bone border [47], large cranial defect [27], delay of cranioplasty [5, 11, 37], and the presence of a ventriculoperitoneal shunt [33]. However, conflicting results from other studies still exist. For instance, Korhonen et al. [29] and Bowers et al. [4] found no significant association between BFR and the time interval between decompressive craniectomy and cranioplasty. A Turkish study did not identify a statistically significant link between defect size and BFR risk [47]. Additionally, evidence from Vietnamese cohorts remains limited. Therefore, we conducted this study to identify associated factors of bone flap resorption following autologous cranioplasty for patients with traumatic brain injuries in Vietnam.

## Methods

### Design and setting

A matched case–control study was conducted analyzing patients who underwent autologous cranioplasty following decompressive craniectomy for traumatic brain injury at Viet Duc University Hospital between January 2022 and July 2025. Cases were patients who developed aseptic bone flap resorption requiring revision cranioplasty by July 2025. Infectious cases were excluded from the study through clinical, radiological examination and microbiological analysis of the bone flap, surrounding tissue specimens and fixation materials obtained during revision surgery. Controls were patients who underwent autologous cranioplasty and showed no clinical or imaging evidence of bone flap resorption throughout the follow-up period.

The case–control ratio was 1:2, with matching criteria including the time of cranioplasty and follow-up duration. Controls had to be operated on the same or the nearest day as their matched cases and must have had a follow-up period that was equivalent or longer than that of the corresponding cases.

### Bone flap preservation and cranioplasty surgery

After decompressive craniectomy, the bone flaps were immediately sent to the hospital tissue bank for a sterile cryopreservation process. They were first cleared of blood and soft tissue, then placed in the biomedical freezer at −80 °C with continuous temperature monitoring. During storage, routine quality checks were carried out and any bone flaps showing signs of contamination were excluded from later reimplantation. On the day of cranioplasty, the bone flaps were defrosted and had the final assessment before implantation.

Cranioplasty was conducted in an elective setting by qualified attending neurosurgeons. The same skin incision as in the decompressive craniectomy was used and the underlying fascia and muscle layers were dissected to expose the skull defect requiring cranioplasty. The bone flap retrieved from the tissue bank was reimplanted into the cranial defect and fixed by non-absorbable sutures or titanium miniplates and screws. A subgaleal drain was routinely placed and the wound was closed in anatomical layers. Postoperative CT imaging was taken within 48 h to detect early complications and the surgical drains were generally removed on postoperative day three. The first postoperative CT scan were also be used as a reference to assess later bone resorption complications.

### Bone flap resorption identification

After CP, the integration and stability of the implanted bone flap were assessed through physical examination and head CT imaging. Clinical signs such as palpable skull defects and localized softening or visible sinking of the flap were suggestive of bone resorption. Follow-up CT scans were compared with immediate postoperative imaging, which served as the radiological baseline. BFR was classified into type I, defined as partial thinning and localized resorption of the bone flap without complete structural collapse or type II in the presence of complete osteolysis involving both the inner and outer tables [10].

### Variables and data collection

Data was extracted from the hospital computer system including patient medical records during hospitalization and follow-up examination notes. These documented all clinical evaluations, imaging results, surgical reports and treatment processes. Regarding demographic and general health features, we investigated age, sex (male or female), body mass index (BMI), current smoking status (during the past 3 months), current alcohol use (units per day in the past 3 months), and comorbidities. Trauma history was also collected, including the mechanism of injury and post-craniectomy complications. Examined peri-cranioplasty characteristics were preoperative Glasgow Outcome Scale Extended (GOSE) score, time interval between DC and CP, bone flap features (size, fragmentation and presence of frontal sinus fracture) and the fixation method of the bone flap (plates and screws or sutures). Using Computed Tomography results, the surface area of the bone flaps was calculated by the elliptic formula (a x b × 3.14/4), in which (a) and (b) were vertical and horizontal diameters, respectively. Post-cranioplasty complications were analyzed with respect to epidural fluid collection (thickness greater than 10 mm) and surgical site infection.

### Data analysis

Continuous variables were presented as means and standard deviations and compared between cases and controls using t-tests. The categorical variables were reported as percentages and group differences were assessed by Chi-square tests and Fisher's exact tests. Conditional logistic regression models were used to analyze the association of variables and the odds of having bone flap resorption. Variables that yielded a *p*-value < 0.2 in univariate analysis were included in the multivariate adjusted logistic regression model. Results are presented as odd ratios with 95% confidence intervals. Statistical analyses were performed using Stata 17.0 (College Station, TX: StataCorp LP).

### Ethical considerations

This study was approved by the Institutional Review Board at Viet Duc University Hospital. Written informed consent was provided by the patients and family members who participated in this study.

## Results

**Fig. 1:**
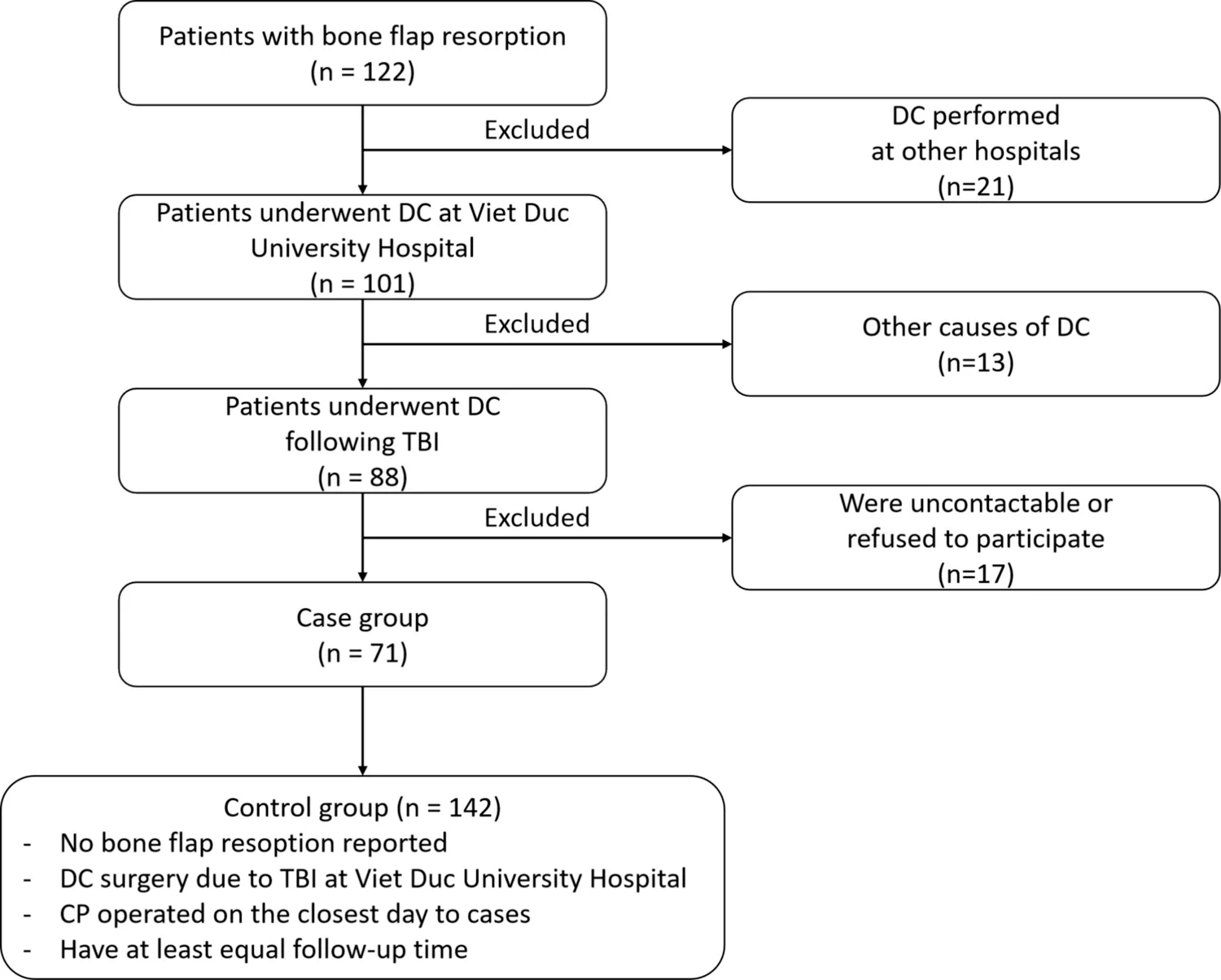
Participant flow chart

In the period from January 2022 to July 2025, 3549 patients underwent autologous cranioplasty in our institution. Among those, 122 patients developed severe bone flap resorption requiring revision. The final study population included 71 cases and 142 controls that fulfilled the inclusion and exclusion criteria (Fig. 1). The case group was comprised of 56 (78.9%) males and 15 (21.1%) females with a mean age of 36.8 ± 15.2 while the control group had 113 (79.6%) males and 29 (20.4%) females with a mean age of 40.4 ± 17.6 (Table 1). Traffic accidents were the cause of TBI in the majority of cases (80.3%), followed by falls (18.8%) and violence (0.9%).

**Table 1:** Univariate analysis of associations between demographic, general health characteristics, trauma history and bone flap resorption

	Case (*n* = 71) *n* (%)	Control (*n* = 142) *n* (%)	*p*-value
Age < 18	14 (19.7)	17 (12.0)	0.13
Sex			
Male	56 (78.9)	113 (79.6)	0.91
Female	15 (21.1)	290.13 (20.4)	
BMI > 25	8 (11.3)	14 (9.9)	0.75
Current smoke	8 (11.3)	12 (8.5)	0.51
Alcohol (> 2 unit/day)	11 (15.5)	18 (12.7)	0.57
Comorbidities			
Diabetes	4 (5.6)	11 (7.8)	0.57
Hypertension	19 (26.8)	36 (25.4)	0.83
Dyslipidemia	1 (1.4)	3 (2.1)	0.59
Chronic hepatitis B or C	10 (14.1)	8 (5.6)	0.04
Chronic kidney disease	0 (0.0)	3 (2.1)	0.29
TBI cause			
Traffic accident	60(84.5)	111(78.2)	0.35
Fall	10(14.1)	30(210.04 0.3)	
Violence	1(1.4)	1(0.7)	
Complications after DC			
Meningitis	6(8.5)	13(9.2)	0.54
Cerebrospinal fluid leakage	2(2.8)	3(2.1)	0.54
Surgical site infection	0(0.0)	1(0.7)	0.67
Ventriculoperitoneal shunt	9(12.7)	7(4.9)	0.04
Pneumocephalus	5(7.0)	8(5.6)	0.69

**Fig. 2:**
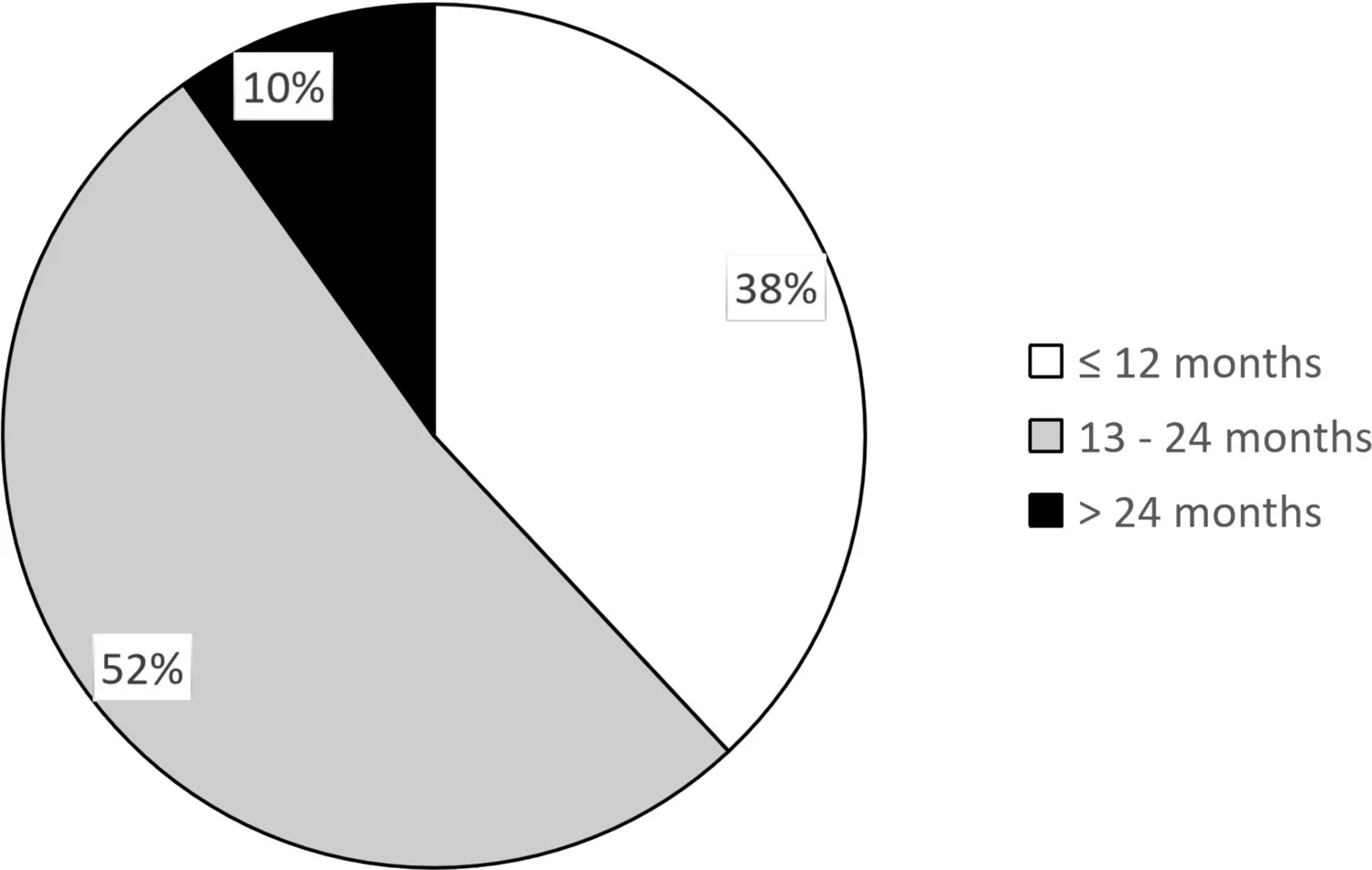
Time interval from cranioplasty surgery to bone flap resorption

A majority of severe bone resorption (Type II) cases occurred in the first and second postoperative years with 27 (38%) and 37 (52%) cases respectively (Fig. 2). The mean time interval from cranioplasty surgery to diagnosis was 444 ± 212 days ranging from 180 to 910 days.

Demographic and general health characteristics and trauma history in the case and control groups are illustrated in Table 1. Patients with aseptic bone flap resorption were more frequently younger than 18 years (19.7% vs. 12.0%, *p* = 0.13) and exhibited a higher prevalence of chronic hepatitis (14.1% vs. 5.6%, *p* = 0.04). Ventriculoperitoneal shunt implantation was also more common in the case group (12.7% vs. 4.9%, *p* = 0.04). No significant differences were observed regarding body mass index, smoking status, alcohol abuse, comorbidities, TBI etiology, or other post-decompressive craniectomy complications.

Perioperative features of the two groups and univariate analysis were presented in Table 2. Larger bone flap size (16.9% vs. 7.8%, *p* = 0.04) and fragmented bone flaps (29.6% vs. 7.8%, *p* < 0.01) were significantly associated with bone flap resorption. On preoperative CT-scan imaging, the rate of frontal sinus fracture was also higher in the case group and showed a trend toward significance (7.0% vs. 2.1%; *p* = 0.07).

**Table 2:** Univariate analysis of associations between peri-cranioplasty characteristics and bone flap resorption

	Case (*n* = 71) *n* (%)	Control (*n* = 142) *n* (%)	*p*-value
GOSE score before cranioplasty (> 4)	55 (77.5)	111 (78.2)	0.91
Late cranioplasty (> 180 days)	5 (7.0)	7 (4.9)	0.53
Bone flap size (> 110 cm^2^)	12 (16.9)	11 (7.8)	0.04
Site of DC			
Unilateral hemicraniectomy	64 (90.1)	119 (83.8)	0.52
Bifrontal craniectomy	4 (5.6)	20 (14.1)	
Bilateral hemicraniectomy	3 (4.2)	3 (2.1)	
Fragmented bone flap	21 (29.6)	11 (7.8)	< 0.01
Frontal sinus fracture	5 (7.0)	3 (2.1)	0.07
Bone flap fixation			
Screws and plates	63 (88.7)	121 (85.2)	0.48
Suture	8 (11.3)	21 (14.8)	
Postoperative complications			
Epidural fluid collection	3 (4.2)	3 (2.1)	0.38
Surgical site infection	0 (0.0)	0 (0.0)	

No significant differences were observed in other variables, including preoperative GOSE scores, timing of cranioplasty, decompressive craniectomy site, epidural fluid collection and surgical site infection following cranioplasty.

Variables with a univariate *p*-value < 0.20 were included in the multivariate model (Table 3). In multivariate analysis, large bone flap size (OR 3.96, 95% CI 1.86–8.43), fragmented bone flaps (OR 7.84, 95% CI 1.38–44.53) and chronic hepatitis (OR 4.32, 95% CI 1.54–12.15) remained independently associated with aseptic bone flap resorption. Although young age, frontal sinus fracture and ventriculoperitoneal shunt implantation were associated with bone flap resorption in the univariate analysis, these associations did not remain significant in the multivariate model.

**Table 3:** Multivariate logistic regression analysis of risk factors for severe aseptic bone flap resorption

Factors	Odds Ratio	95% CI	*p*-value
Young age	1.90	0.79–4.57	0.15
Hepatitis	3.30	1.06–10.24	0.04
Large bone flap size	3.09	1.05–9.08	0.04
Fragmented bone flap	5.28	2.04–13.65	< 0.01
Frontal sinus fracture	3.31	0.65–16.94	0.15
Ventriculoperitoneal shunt	2.00	0.63–6.33	0.24

## Discussion

Cranioplasty using autologous bone is considered a special form of autologous transplantation because the bone flap has typically been cryopreserved and most of its cellular components are non-viable at the time of reimplantation [3, 6]. The reintegration of the bone flap greatly depends on the balance between the osteoblastic bone formation and osteoclastic bone resorption [19, 42, 45, 50]. When osteoclastic activity predominates, the flap progressively thins, ultimately leading to cranioplasty failure [19].

BFR is a slow and progressive process that emerges shortly after reimplantation. Although early radiological signs may be detectable on CT scans within a few months, significant resorption requiring revision is often diagnosed much later [48]. In our cohort, BFR-related cranioplasty failure occurred at a mean of 444 ± 212 days (approximately 14.8 ± 7.0 months) after surgery, which is comparable to findings from previous studies. Schuss et al. reported a mean time to BFR-related cranioplasty failure of 20± 17 months [40], whereas Honeybul et al. observed most cases approximately 12 months after surgery [25].

In the present study, fragmented bone flaps, large flap size, and chronic hepatitis were identified as independent predictors of postoperative bone resorption.

### Fragmented bone flap

Fragmentation often occurs in the setting of high-energy head trauma or when the bone flap is removed in multiple pieces during decompressive craniectomy. In our study, patients with fragmented flaps exhibited a significantly higher incidence of bone flap resorption compared to those with single-piece flaps, which is consistent with prior reports [4, 5, 10, 43]. in a retrospective study of 372 patients, reported that bone flaps broken into two pieces increased the odds of necrosis by 3.35-fold, whereas fragmentation into three or more pieces raised the risk by 24-fold compared with intact flaps [10]. Brommeland et al. also found flap fragmentation as a strong independent predictor of bone flap resorption, yielding an odds ratio of 14.3 (*p*= 0.005) [5].

In cryopreserved autografts, which lack viable osteoblasts, survival depends on revascularization and contacts of bone with dura and periosteum [18]. Fragmentation creates gaps, thereby impeding vascular invasion and osteoprogenitor cell migration to the bone flap, ultimately preventing osseous union with the native calvarium [5]. Moreover, unstable fragments permit subtle micromotion under the scalp, which continuously stimulates bone remodeling [2].

Careful surgical planning during decompressive craniectomy may reduce the need to remove the bone flap in multiple fragments. However, when extensive fragmentation is unavoidable, the use of synthetic materials for subsequent cranioplasty should be considered because of the increased risk of BFR.

### Bone flap size

In this study, bone flap size (≥ 110 cm^2^) was identified as a risk factor of BFR after autologous cranioplasty. This finding aligns with the study by Schoekler and Trummer, concluding that large bone flaps were more susceptible to BFR [39]. Similarly, in a retrospective cohort study, Kim et al. also reported an increased risk of resorption in patients with bone flaps larger than 110 cm^2^ [27].

Many theories have been hypothesized to explain the impact of this factor on the survival of the implanted flap. After the cryopreservation period, larger bone flaps tend to accumulate greater amounts of devitalized and necrotic tissue, which amplifies local inflammatory responses. Proinflammatory cytokines such as tumor necrosis factor alpha (TNF-α) and interleukin 1 beta (IL-1β) stimulate osteoclast differentiation and activity while suppressing osteoblast function [38, 44]. In addition, the reconstruction of a wide cranial defect is often associated with greater soft-tissue disruption and a higher likelihood of vascular injury, both of which impair neovascularization and bony reintegration [26].

Although a large decompressive craniectomy is of pivotal importance for effective intracranial pressure control in emergency settings, the risk of BFR may be reduced by proper surgical techniques. Preservation of the main vessel of the scalp as well as careful handling of soft tissue should be achieved [17].

### Viral hepatitis

In the present study, 8.5% of patients had chronic viral hepatitis (HBV or HCV), partially reflecting the high prevalence of viral hepatitis in Vietnam. Although HCV infection affects approximately 1% of the Vietnamese population, comparable to the global estimate of 0.7%, HBV is substantially more common, affecting approximately 10.5% of the population, which markedly exceeds the global average of 3.3% [12, 22].

Chronic viral hepatitis was associated with an increased risk of bone flap resorption after cranioplasty in our cohort. To our knowledge, this association has not been previously reported in the context of cranioplasty outcomes; however, the connection between chronic viral hepatitis and bone health has been established. Hepatitis, particularly in its chronic viral forms (HBV or HCV), is reported to increase the risk of osteoporosis [7, 20, 21, 30, 35]. The presence of hepatitis viruses can be a chronic trigger of cytokine release. The circulating cytokines, especially tumor necrosis factor (TNF), might promote osteoclastic activity inside the bone mineralized matrix, thereby accelerating bone resorption [16, 34, 49]. Moreover, viral hepatitis is also linked to the decrease in vitamin D [20, 24] and insulin-like growth factor 1 [13] as well as the dysregulation in the RANK–RANKL–OPG signaling pathway [32], all of which further disrupt bone remodeling homeostasis.

Given that successful cranioplasty relies on revascularization, osteoblast survival, and balanced bone remodeling, the systemic pro-resorptive environment observed in chronic viral hepatitis may compromise bone flap incorporation and accelerate resorptive processes. Antiviral treatments, particularly nucleotide analogues such as tenofovir, have been associated with reduced bone mineral density and may further contribute to bone fragility [1, 15, 46]. Unfortunately, treatment status and duration of infection could not be fully evaluated in the present study.

Future prospective studies incorporating bone metabolic markers, vitamin D levels, liver function indices and antiviral treatment data are needed to clarify causality and to identify high-risk patients who may benefit from alternative reconstructive strategies.

Several studies have identified younger age and ventriculoperitoneal shunt implantation as potential risk factors for bone flap resorption following autologous cranioplasty [8, 33, 43]. In our study, both factors were associated with severe aseptic bone flap resorption in the univariate analysis but did not remain statistically significant after multivariable adjustment. The lack of an independent association in our study may be attributable to the limited number of younger and ventriculoperitoneal-shunted patients.

### Strengths and limitations

This study benefits from the single-center design which ensured consistent clinical practice and radiological assessment throughout the study period, thereby reducing potential confounding related to differences in practice patterns. However, the relatively small sample size may have limited the statistical power to detect significant associations. The retrospective case–control design is subject to potential information bias and can not establish causality. Furthermore, the inclusion of only severe cases requiring revision surgery may limit the generalizability of the findings to patients with milder forms of bone flap resorption. Further prospective, multicenter studies are warranted to validate these findings and clarify the risk factors for bone flap resorption following autologous cranioplasty.

## Conclusions

Bone fragmentation, bone flap size, and chronic viral hepatitis were associated with a higher rate of bone flap resorption after cranioplasty. Further studies are necessary to confirm these findings, especially those concerning the role of chronic viral hepatitis.

## Data Availability

No datasets were generated or analysed during the current study.

## Abbreviations

TBI: Traumatic brain injury
CP: Cranioplasty
DC: Decompressive cranioplasty
BMI: Body mass index
GOSE: Glasgow Outcome Scale – Extended
BFR: Bone flap resorption
OR: Odds ratio
CI: Confidence interval
HBV: Hepatitis B virus
HCV: Hepatitis C virus
